# Intravenous Thrombolysis in Posterior Circulation Stroke

**DOI:** 10.3389/fneur.2019.00417

**Published:** 2019-04-26

**Authors:** Tomáš Dorňák, Michal Král, Daniel Šaňák, Petr Kaňovský

**Affiliations:** Department of Neurology, Palacky University and University Hospital, Olomouc, Czechia

**Keywords:** stroke, posterior circulation, intravenous thrombolysis, intracranial hemorrhage, ischemia

## Abstract

**Background:** Intravenous thrombolysis (IVT) is a standard treatment for both anterior circulation ischemic stroke (ACIS) and posterior circulation ischemic stroke (PCIS). PCIS is a clinical syndrome associated with ischemia-related changes in the territory of the posterior circulation arteries. Embolism is the most common stroke mechanism in posterior circulation. PCIS represents 12–19% of all IVT-treated strokes.

**Methods and Results:** We searched the PubMed database for assessments of intracerebral hemorrhage (ICH) and clinical outcome in PCIS patients treated with IVT. ICH occurs in 0–6.9% of posterior ischemic stroke depending on the definition of symptomatic ICH, and any ICH in 17–23.4% of posterior ischemic stroke. For patients with PCIS, 38–49% have a favorable outcome (mRS 0–1) after IVT. Better clinical outcomes occur more often in patients with PCIS than in those with ACIS. The mortality rate among PCIS patients treated with IVT ranges from 9 to 19%; it does not differ significantly between PCIS and ACIS.

**Conclusions:** Up to date, no data about PCIS and IVT are available from RTCs. Based on limited results from retrospective clinical studies and case series, IVT is safer for use in PCIS than in ACIS. Patients with brainstem ischemia, vertebral artery occlusion, and absence of basilar or posterior cerebral artery occlusion could be considered for treatment with IVT even in borderline cases. Time to IVT in PCIS seems to be a less crucial factor than in ACIS. IVT for PCIS may be beneficial even after 4.5 h from symptom onset.

## Introduction

### History of Intravenous Thrombolysis—The Most Relevant Studies

Intravenous thrombolysis (IVT) is a standard treatment for both anterior circulation ischemic stroke (ACIS) and posterior circulation ischemic stroke (PCIS). Recombinant tissue plasminogen activator (rtPA, alteplase) was licensed for the first time in 1996 in North America for intravenous use within 3 h. A restricted conditional license for the use of rtPA within 3 h was granted in Europe in 2002. At the beginning of the rtPA era (1992/1993), three smallplacebo-controlled studies reported its efficacy in the early stages of ischemic stroke (1–3).

Later, three much largerplacebo-controlled trials showed the benefits of intravenous rtPA given to patients with ischemic stroke selected by clinical symptoms and CT. Studies by the National Institute of Neurological Disorders and Stroke (NINDS) and Alteplase Thrombolysis for Acute Noninterventional Therapy in Ischemic Stroke A + B (ATLANTIS) demonstrated improvements in functional outcomes at 3 months if thrombolysis was administered within 3 h of symptom onset, with the greatest benefit seen within 90 min from symptom onset (4, 5). The European Cooperative Acute Stroke Study (ECASS) III trial tested and proposed improvements in clinical outcome after intravenous alteplase administered between 3 and 4.5 h (6). Five other placebo-controlled trials showed no clear benefit of early rtPA treatment (7–11).

All these studies were done on patients up to 80 years old with a specific type of stroke. The International Stroke Trial (IST 3), a placebo-controlled trial, was initiated to provide evidence for a wide range of patients (12). It reported improved functional outcome up to 6 h on 3,035 patients. The benefit did not seem to be diminished in elderly patients. In the rtPA group, 37% of patients were alive and independent, with a 7% risk for symptomatic intracranial hemorrhage (sICH); in the control group, 35% of patients were alive and independent, with a 1% risk for sICH. These questionable results summarized in Table 1 led to greater pressure on patient selection and a search for outcome and sICH predictors.

**Table 1:** History of intravenous thrombolysis in randomized controlled trials.

	**Year**	**rtPA dose**	**Number of patients**	**Age range**	**Time window**	Exclusion CT criteria^*^	**Follow up**	Overall risk-benefit^***^	**rtPA-favorable outcome**	**Placebo-favorable outcome**	**rtPA-all ICH**	**Placebo-all ICH**	**rtPA-sICH**	**Placebo-sICH**
ECASS I	1995	1.1 mg/kg	620	18–80 y	0–6 h	Diffuse swelling, parenchymal hypodensity, ischemia in more than 1/3 of MCA territory	3 months	–	35.7%	29.3%	42.8%	36.8%	–	–
ECASS II	1998	0.9 mg/kg	800	18–80 y	0–6	Swelling exceed 1/3 of MCA territory	3 months	–	40.3%	36.6%	48.4%	40.2%	2.5 more SICH//	
ECASS III	2008	0.9 mg/kg	821	18–80 y	3–4.5	Major ischemic infarction	3 months	+	52.4%	45.2%	27.0%	17.6%	2.4%	0.3%
NINDS	1995	0.9 mg/kg	624	18–80 y^**^	0–3	None	3 months	+	39%	26%	–	–	12%	2%
EPITHET	2008	0.9 mg/kg	101	18–80 y^**^	3–6	infarction >1/3 MCA territory	3 months	–	36%	21%	–	–	7.7%	0%
ATLANTIS A	2000	0.9 mg/kg	142	18–80 y	0–6	None	3 months	–	No difference on day 90//		–	–	8%	0%
Atlantis B 3–5	1999	0.9 mg/kg	613	18–80 y	3–5	Infarction >1/3 MCA territory	3 months	–	34%	32%	–	–	7.0%	1.1%
Atlantis A + B 0–3	2001	0.9 mg/kg	61	18–80 y	0–3	As for ATLANTIS A and B	3 months	+	61.1%	45.5%	–	–	13%	0%
IST 3	2012	0.9 mg/kg	3035	18 + (53% > 80 y)	0–6	None	6 months	+	37%	35%	–	–	7%	1%

* Intracranial hemorrhage were excluded in all studies.

** 69 NINDS patients and 25 EPITHET patients were older than 80.

*** Judged by authors themselves in the article.

//Exact numbers not available.

*ICH, intracranial hemorrhage; MCA, middle cerebral artery; rtPA, recombinant tissue plasminogen activator; sICH, symptomatic intracranial hemorrhage*.

### Definition of PCIS

PCIS is a clinical syndrome associated with ischemia related to stenosis, *in situ* thrombosis, or embolic occlusion of the posterior circulation arteries (13). Posterior circulation arteries comprise the territory of the vertebral, cerebellar, posterior cerebral, and basilar artery. ACIS and PCIS differ in several respects: frequency, symptoms and signs, etiology, risk for recurrent stroke, sensitivity, and specificity of diagnostic modalities and prehospital screening instruments, acute management, complication after acute treatment, and clinical outcome (13–20).

### Anatomy and Clinical Features

Posterior circulation comprises the territory of the vertebral arteries, basilar artery, and posterior cerebral arteries. Vertebral arteries and their branches supply blood to the medulla and cerebellum. They are often asymmetric and one is dominant, causing an elongation of the basilar artery to the opposite site (21). The basilar artery is joined to the brainstem by penetrating the caudal, middle, and rostral branches that form anastomoses in 42 to 67% of the cases (22). Proximal and middle segments, including the anterior inferior cerebellar artery and the superior cerebellar artery, supply the pons and cerebellum; distal segments supply the mesencephalon. The apex of the basilar artery supplies the median and paramedian portions of the midbrain and thalamus. Posterior cerebral arteries supply oxygenated blood to the temporal and occipital lobe, part of the thalamus, the walls of the third ventricle, choroid plexus, cerebral peduncle, fornix, and caudate nucleus (23).

The posterior circulation is rich in collaterals and clinical manifestations of disturbed flow are therefore highly variable (22, 24). In addition, symptoms and signs considered typical for PCIS occur far less often than was expected. Inaccurate localization would occur commonly if clinicians relied on the clinical neurological deficits alone (17, 19). Among 407 New England Medical Center Posterior Circulation registry patients, the most common symptoms were dizziness (47%), unilateral limb weakness (41%), dysarthria (31%), gait ataxia (31%), headache (28%), nausea or vomiting (27%), and nystagmus (24%) (13, 24). Numerous eponyms are linked to posterior circulation stroke syndrome, many of which occur in an incomplete form (25). Diverse clinical symptoms of PCIS potentially contribute to a delay in diagnosis. Burns et al. (26) reported the median time from emergency department arrival to diagnosis as 8 h 24 min for basilar artery and 1 h 23 min for left middle cerebral artery.

### Pathophysiology and Causes

In a large single-center study on 407 patients, embolism was the commonest stroke mechanism (40% of patients including 24% cardiac origin, 14% intra-arterial, and 2% cardiac and arterial sources) (24). Cerebellar infarct without concomitant brainstem or occipital infarct was associated with cardioembolism (67%); isolated paramedian pontine or midbrain infarct was associated with basilar artery stenosis (71%) (27). Vertebral artery dissection is a rare cause of stroke in the general population; however, it represents one of the more common causes of stroke in patients younger than 45 years of age. Neck distortions such as chiropractic manipulation, bending of the neck, and blunt trauma cause the dissection (28).The etiology of PCIS appears to be different in non-Caucasians, with a rare (5.2%) chance of cardioembolism (27).

### Diagnostic Methods

The recognition of stroke in the prehospital phase and in the emergency room can be done by the Face Arm Speech Test (FAST), ABCD 2, and Recognition of Stroke In the Emergency Room (ROSIER) scales. Both FAST and ABCD2 scores, which have been developed as screening tools for unselected strokes, are less effective in the diagnosis and identification of high-risk cases for PCIS and transitory ischemic attack (TIA) (14).The ROSIER scale seems to be more sensitive to marking PCIS as a potential stroke, because this scale includes a visual field defects evaluation (29). Although still giving more weight to symptoms of anterior circulation stroke, the National Institute of Health Stroke Scale (NIHSS) scale does include PCIS specific symptoms like gait ataxia and visual field loss. It is frequently used in daily clinical practice to categorize severity of stroke. The expanded NIHSS (e-NHISS) might improve the sensitivity of NIHSS for PCIS symptoms (30).

All cases of suspected stroke must be further diagnosed with CT or magnetic resonance imaging (MRI) to exclude intracranial hemorrhage (ICH). If a patient is a candidate for IVT, then vessel imaging should be done without delay to exclude large vessel occlusion. Acute phase CT imaging is generally more available than MRI. MRI is similar to CT in detecting acute ICH (31). Diffusion-weighted imaging (DWI) done within 3 h can detect ischemic changes with 73–92% sensitivity and near 100% in the first 6 h after symptom onset (32). MRI is therefore superior to non-contrast CT, which has sensitivity below 20% within first 3 h and only 57–71% in the first 24 h after onset of stroke (31, 32). Thus, MRI can help to diagnose disorders that mimic stroke and TIA more accurately. Despite the high sensitivity of MRI, a false-negative DWI result can happen during the first 24 h of PCIS. Posterior ischemic stroke stroke should therefore not be excluded on the basis of early negative DWI, especially when vertebrobasilar suggestive symptoms persist (18).

CT angiography (CTA) and time of flight (TOF) magnetic resonance angiography (MRA) have high sensitivity for intracranial stenosis (98 and 70%, respectively) and occlusion (100 and 87%, respectively). CTA offers better diagnostic accuracy than TOF MRA and is recommended over TOF MRA for detecting intracranial stenosis and occlusion. CTA has a high interoperator reliability and is superior to DSA in the evaluation of posterior circulation steno-occlusive disease when slow flow is present (33).

Contrast-enhanced MRA has a higher sensitivity than TOF-MRA and with similar sensitivity to DSA in ischemic stroke patients. Contrast-enhanced MRA is superior to TOF-MRA in localizing vessel occlusion and assessment of collateral status, providing a larger coverage (extracranial vessels) with shorter acquisition time (34).

In a study of 436 patients with PCIS who underwent perfusion computed tomography (CTP), multiple clinical, etiological, and radiological variables were associated with focal hypoperfusion (20). Focal hypoperfusion was associated with a worse 12-month outcome (20).

## IVT in Posterior Circulation Stroke

### Frequency of PCIS in IVT Trials

PCIS represents 12–19% of all IVT-treated strokes (16, 35, 36). Patients with PCIS can could not significantly influenced the results of randomized controlled trials, given that PCIS patients were under-represented in these studies: 5% of the PCIS patients in the NINDS study and 0% in the ECASS I and II trials; and there is no information on PCIS representation is available from the ATLANTIS and the ECASS III trials (4–10).

In most of the published studies, stroke territory was classified according to clinical presentation in combination with CT and MRI findings. Proportion of CT and MRI done was not mentioned in most of them (35–37) or only CT findings were taken into account (38). In Dorňák et al. study, control imaging by CT/MRI/none was done on 30/68/2 PCIS patients, respectively, and on 436/329/12 ACIS patients, respectively (16).

### Clinical Outcome

There are some placebo-controlled trials that reported positive effects and benefits of IVT on clinical outcome in ACIS patients (4–6, 12). No data on efficacy of IVT in PCIS are available from randomized control studies. Based on the results of only retrospective clinical studies, patients with PCIS had 38–49% favorable outcomes (mRS 0–1) after IVT (16, 36).

Better clinical outcomes after 3 months occur more often (66 vs. 47% respectively, *P* < 0.001) as well as lower NIHSS scores after 2 and 24 h in patients with PCIS than in those with ACIS (35, 36). Nevertheless, posterior circulation territory is not associated with favorable outcome after multivariable adjustments in all cause ischemic strokes (36).

Blood glucose level, NIHSS score, age, antiplatelet medication, and anticoagulation are independently associated with a favorable outcome. The initial NIHSS score seems to be less important predictor of outcome than decreased consciousness measured by the Glasgow Coma Scale (39). Mortality rate among PCIS patients treated with IVT ranges from 9 to 19% (16, 36, 37) and even though it is lower in PCIS, it does not differ significantly between PCIS and ACIS (36).

### Definitions and Frequency of ICH Following IVT

It is well-known that risk might outweigh benefit beyond 4.5 h in ACIS. Intracranial hemorrhage is the most feared complication of IVT. It occurs in 0–6.9% of patients with PCIS, depending on the definition of symptomatic ICH, and any ICH in 17–23.4% of patients with PCIS (16, 35–37). Table 2 shows various definitions ofsICH. The ECASS 2 and SITS-MOST sICH definitions are said to best identify tPA hemorrhages that alter final patient outcome (40). However, the definition of “symptomatic” raises several concerns. PCIS symptoms are not well-represented by the NIHSS, which is frequently used to categorize ICH (41). In addition, sICH is a subjective term with clinical deterioration that could occur for various other reasons and may be evaluated differently by different physicians or missed if a patient is in coma or mechanically ventilated. For many reasons, symptomatic definitions are not suitable for retrospective studies without tight protocol documentation.

**Table 2:** Various definitions of symptomatic intracerebral hemorrhage.

	**Clinical**	**Radiological**
NINDS	Any	Any
ECASS 2	Deterioration, adverse events, or ≥4 NIHSS	Any
ECASS 3	≥4 NIHSS	Any
SITS-MOST	≥4 NIHSS	Parenchymal hemorrhage 2

*NINDS: any ICH not seen on a previous CT scan and subsequently either a suspicion of hemorrhage or any decline in neurologic status. ECASS 2: any ICH with clinical deterioration, or adverse events indicating clinical worsening (e.g., drowsiness, increase of hemiparesis) or causing a decrease in the NIHSS score of 4 or more points. ECASS 3: any ICH associated with clinical deterioration, as defined by an increase of 4 points or more in the score on the NIHSS, or that led to death. SITS-MOST: local or remote parenchymal hemorrhage type 2 on the 22–36 h post-treatment imaging scan, combined with a neurological deterioration of 4 points or more on the NIHSS from baseline, or from the lowest NIHSS value between baseline and 24 h, or leading to death*.

In a study by Sarikaya et al. (36) on 883 consecutive acute stroke patients (out of which 95 were PCIS patients) treated with IVT in three Swiss stroke centers, there were a total of 36 (4%) sICH patients according to NINDS criteria. Patients with PCIS had sICH less often (0 vs. 5%, *P* = 0.026) and PCIS was an independent predictor of a lower frequency of sICH. Another study on 84 PCIS patients demonstrated a trend of increasing incidence of ICH across various types of sICH (*P* = 0.0001 per NINDS, *P* = 0.001 per ECASS II, *P* = 0.002 per SITS-MOST, and *P* = 0.008 for any ICH (35). In a single-center study by Dorňák et al. (16) with a similar number of patients, ICH according to ECASS I criteria (independent of clinical manifestation) was significantly less frequent in PCIS than in ACIS patients (5.1 vs. 17.2%, respectively). The risk for ICH was 3.4 times higher in ACIS than in PCIS. In addition, the risk for large hemorrhage (PH1 + PH2) was 5.2 times greater in ACIS. No significant difference was observed between ACIS and PCIS for small petechial hemorrhage (HI1 + HI2 per ECASS I).

The reason for low frequency of ICH in PCIS is unknown and rather hypothetical. A smaller lesion volume in infratentorial strokes (42) and better collateral circulation in comparison with the middle cerebral artery (43) as well as the fact that the brainstem is nourished by small end arteries might partly explain the lower sICH occurrence. In addition, pretreatment blood-brain barrier derangement is an infrequent finding in acute PCIS and may be associated with an increased risk of parenchymal hemorrhage development in patients undergoing recanalization therapy (44).

The lower risk of IVT in PCIS could suggest that the benefit of IVT administration, even beyond 4.5 h or in borderline cases, may outweigh the potential risk. Borderline cases refer to patients who are relatively indicated/contraindicated because the class (strength) of recommendation is Class IIb or III according to the most recent AHA/ASA recommendations from 2018. Those are the situations where the benefit = or ≥ the risk (45). The knowledge of ICH predictors following IVT is thus extremely useful.

### ICH Predictors After IVT in PCIS

Various risk score models have been developed to predict sICH after IVT, but due to the generally low occurrence of PCIS, such scoring systems are mostly designed and tested for ACIS (46, 47).

In a study by Sarikaya et al. (36), atrial fibrillation (*P* = 0.019), antiplatelet medication (*P* = 0.025), and diastolic blood pressure (*P* = 0.029) were identified using multivariate logistic regression analyses as independent predictors of sICH according to NINDS criteria. Nevertheless, due to the lack of sICH in PCIS (0%), no predictors for PCIS could be identified. Undermined logistic regression analyses by the low occurrence of sICH was a problem in the most of published studies with PCIS patients (16, 35–37, 48). The largest study by Dorňák et al. (49) on 158 PCIS patients has enough patients to identify predictors of ICH in PCIS. Atrial fibrillation (*p* = 0.004), NIHSS score at time of treatment (*p* = 0.016), decreased level of consciousness (*p* = 0.003), basilar artery occlusion (*p* = 0.007), occlusion of PCA (*p* = 0.001), and additional endovascular therapy (*p* = 0.001) were identified as significant predictors for ICH (according to ECASS I) in PCIS (7, 49).

Supratentorial localization of acute ischemia in the territory of the PCA (*p* = 0.025) as well as the bigger volume of ischemic changes in this localization (*p* = 0.025) significantly increase the risk of ICH (49). The brainstem was the only localization out of four established groups (brainstem, cerebellum, thalamus, and supratentorial territory of PCA) that decreased, although statistically not significantly, the risk of ICH (*p* = 0.545) (49).

Time to IVT in PCIS seems to not crucially influence ICH risk or chances for unfavorable outcomes, as it does in ACIS (49, 50). IVT for PCIS may be beneficial when initiated even 8 h after symptom onset (39, 51). Thrombolysis 4.5–6 h after stroke onset reduced infarct growth and increased the rate of reperfusion, which was associated with good neurological and functional outcome (52).

### IVT in Basilar Artery Occlusion

IVT in basilar artery occlusion (BAO) can be used alone or prior to endovascular treatment asa bridging in terms of the “drip, ship, and retrieve” approach. Several studies reported the efficacy of IVT alone, similar to invasive endovascular therapy, reaching a good clinical outcome (mRS 0–2) in 21–53% of the patients and as mRS 0–3 in 26–63% as shown in Table 3 (50). The recanalization rate of IVT alone is 52–78%, and it almost reaches the efficacy of intra-arterial thrombolysis (IAT), which varies from 63 to 94% (50). Data from the Basilar Artery International Cooperation Study (BASICS) on 592 basilar artery occlusion patients do not support unequivocal superiority of IAT over IVT (53). Brandt et al. (54) suggested that occlusion of distal one-third of the basilar artery is associated with a lower mortality rate than occlusion of proximal and/or middle portions of the basilar artery. Despite the fact that thrombus location in the proximal or middle segments of the basilar artery often result in large pontine strokes with severe deficits, no significant association between localization of occlusion and outcome was reported in several studies (53, 55–57).

**Table 3:** Outcome in basilar artery occlusion patients treated with IVT.

**Study**	**Year**	***n***	**Modified Rankin Scale**			**Mortality (%)**	**Time of follow-up**	**SICH (%)**	**Recanalization rate (%)**
			**0–2 (%)**	**0–3 (%)**	**4–5 (%)**				
Lindsberg	2004	50	22	32	28	40	3 months	14	52
		50	30	34	20	46	1 year		
Lindsberg, Mattle^*^	2006	76	22	N/A	N/A	50	Varies	11	53
BASICS—MtM	2009	49	53	63	20	16	1 month	6	71
BASICS—S	2009	72	21	26	28	46	1 month	6	66
Sairanen	2011	116	26	36	22	41	3 months	16	65
Miyagi^#^	2012	25	48	N/A	N/A	4	3 months	8	78

BASICS, Basilar Artery International Cooperation Study; MtM, mild to moderate deficit; n, number; S, severe deficit; SICH, symptomatic intracranial hemorrhage.

* Systematic review of literature up to 2005.

# Patients treated with low-dose alteplase (0.6 mg/kg).

The bridging approach combines the speed of widely accessible IV agents with the high recanalization rate of endovascular techniques. Metanalyses on 15 studies demonstrated that bridging is associated with acceptable safety and efficacy in stroke patients (58). Bridging therapy shortens the time to any specific recanalization treatment, which seems to be the only modifiable independent predictor of both 30-day and 90-day clinical outcome. Thus, additional endovascular treatment should be started as soon as possible and not considered only as a rescue strategy (57, 58). Data from two small series on basilar artery occlusion patients (52, respectively 70 patients) reported better 90-day clinical outcomes in patients treated with bridging (59, 60).

## Conclusion

Up to date, no data about IVT in PCIS are available from RTCs. Based on limited results from retrospective clinical studies and case series, IVT is safer for use in PCIS than in ACIS. Patients with brainstem ischemia, vertebral artery occlusion, and absence of basilar or posterior cerebral artery occlusion could be considered for treatment with IVT even in borderline cases. Those patients seem to experience favorable outcome less frequently despite not having an increase in ICH rates. Time to IVT in PCIS seems to be a less crucial factor than in ACIS. IVT for PCIS may be beneficial even after 4.5 h from symptom onset.

## Author Contributions

TD conceived the presented idea and wrote the first draft of the manuscript. MK, DŠ, and PK provided critical feedback and helped shape the manuscript.

### Conflict of Interest Statement

The authors declare that the research was conducted in the absence of any commercial or financial relationships that could be construed as a potential conflict of interest.
